# Therapeutic Potential of Calcium Channel Blockers in Neuropsychiatric, Endocrine and Pain Disorders

**DOI:** 10.3390/cells14141114

**Published:** 2025-07-20

**Authors:** Aarish Manzar, Aleksandar Sic, Crystal Banh, Nebojsa Nick Knezevic

**Affiliations:** 1 Department of Anesthesiology, Advocate Illinois Masonic Medical Center, Chicago, IL 60657, USA; aarishsyedmanzar@gmail.com (A.M.); aca.smed01@gmail.com (A.S.); crystal.banh@my.rosalindfranklin.edu (C.B.); 2 Department of Internal Medicine, Ziauddin Medical College, Karachi 75500, Pakistan; 3 Department of Anesthesiology, University of Illinois, Chicago, IL 60612, USA; 4 Department of Surgery, University of Illinois, Chicago, IL 60612, USA

**Keywords:** L-type voltage-gated calcium channels, calcium signaling dysregulation, mood stabilization, neuroinflammation, central nervous system pharmacotherapy, drug repurposing, pharmacovigilance

## Abstract

Calcium channel blockers (CCBs), originally developed for cardiovascular indications, have gained attention for their therapeutic potential in neuropsychiatric, endocrine, and pain-related disorders. In neuropsychiatry, nimodipine and isradipine, both L-type CCBs, show mood-stabilizing and neuroprotective effects, with possible benefits in depression, bipolar disorder, and schizophrenia. In endocrinology, verapamil, a non-dihydropyridine L-type blocker, has been associated with the preservation of pancreatic β-cell function and reduced insulin dependence in diabetes. CCBs may also aid in managing primary aldosteronism and pheochromocytoma, particularly in patients with calcium signaling mutations. In pain medicine, α2δ ligands and selective blockers of N-type and T-type channels demonstrate efficacy in neuropathic and inflammatory pain. However, their broader use is limited by challenges in central nervous system (CNS) penetration, off-target effects, and heterogeneous trial outcomes. Future research should focus on pharmacogenetic stratification, novel delivery platforms, and combination strategies to optimize repurposing of CCBs across disciplines.

## 1. Introduction

Calcium channel blockers (CCBs) were first discovered in the 1960s during pharmacological screenings of agents designed to induce coronary vasodilation, marking a turning point in cardiovascular pharmacotherapy [1]. By the 1970s, they entered clinical use in the United States as part of antihypertensive protocols and shortly after gained recognition for their efficacy in treating vasospastic and stable angina [2]. Over the following decades, their use became firmly established within cardiology, especially in the management of hypertension, arrhythmias, and ischemic heart disease.

CCBs are chemically and functionally classified into dihydropyridines and non-dihydropyridines. These groups differ in tissue selectivity, pharmacokinetics, and clinical indications. While dihydropyridines primarily act on vascular smooth muscle and are used in controlling blood pressure and anginal symptoms, non-dihydropyridines exhibit more pronounced effects on cardiac conduction and contractility, making them suitable for arrhythmia management [3].

Mechanistically, all CCBs function by inhibiting L-type voltage-gated calcium channels (Cav1.2 and Cav1.3), thereby reducing intracellular calcium influx, a process vital for vascular tone, cardiac excitability, endocrine secretion, and neuronal signaling [4]. The widespread distribution of L-type calcium channels across various tissues has paved the way for broader therapeutic interest [5]. Calcium signaling plays an important role in the pathophysiology of numerous neuropsychiatric disorders, including anxiety, bipolar disorder, schizophrenia, and neurodegenerative diseases. Dysregulated calcium homeostasis has been implicated in abnormal neurotransmitter release, neuronal hyperexcitability, and synaptic dysfunction, providing a mechanistic rationale for investigating CCBs as neuromodulatory agents [6,7].

In the field of endocrinology, calcium signaling is central to hormone secretion, particularly insulin release from pancreatic β-cells and aldosterone synthesis in the adrenal cortex. Several studies have explored CCBs in the context of type 2 diabetes, insulin resistance, primary hyperaldosteronism, and even polycystic ovary syndrome (PCOS), suggesting a potential role in metabolic regulation [8,9,10,11]. 

In pain medicine, calcium channels have shown potential as mediators in nociceptive transmission, neuropathic pain, and central sensitization. CCBs have been studied for their analgesic properties in conditions such as migraine, complex regional pain syndrome (CRPS), and chemotherapy-induced neuropathy [12,13,14].

This review aims to provide a focused and updated synthesis of therapeutic potentials of CCBs outside cardiology, with specific attention to neuropsychiatric disorders, endocrine dysfunctions, and chronic pain conditions. Although CCBs have historically been investigated for a wide array of conditions, subsequent evidence has often revealed limited efficacy or inconsistent results. Therefore, we examined current literature to identify which emerging indications are most actively being investigated today. We specifically aim to identify the most promising directions for future research by critically evaluating biological plausibility and the current state of evidence across each domain.

**Figure 1:**
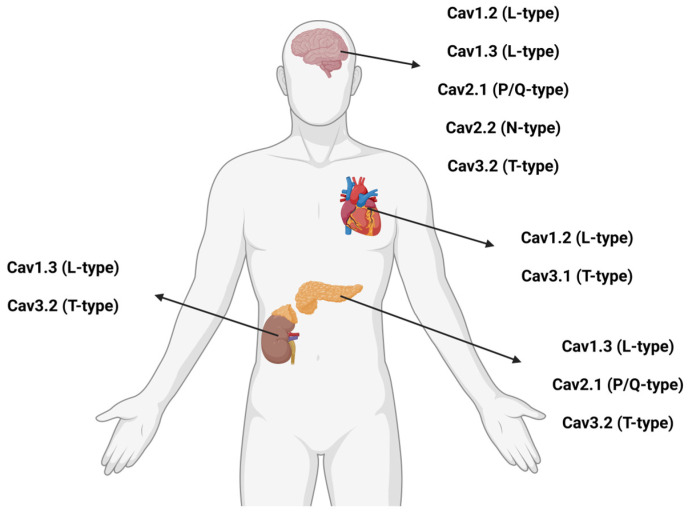
Tissue-specific distribution of voltage-gated calcium channel subtypes. The figure shows predominant expression sites of L-type (Cav1.2, Cav1.3), P/Q-type (Cav2.1), N-type (Cav2.2), and T-type (Cav3.1, Cav3.2) channels across the brain, heart, pancreas, and adrenal gland. Created in BioRender online application, available at https://app.biorender.com/. (Accessed on 17 July 2025).

A schematic overview of tissue-specific distribution of calcium channel subtypes (L-type, P/Q-type, N-type, and T-type) is presented in Figure 1 below to illustrate their relevance across neurological, endocrine, and cardiovascular systems.

## 2. Calcium Channel Blockers in Neuropsychiatric Disorders

### 2.1. Major Depressive Disorder (MDD)

Studies have suggested a potential association between the use of certain CCBs and an increased risk of developing major depressive disorder (MDD), particularly in comparison with diuretics [15]. However, brain-penetrant agents like nifedipine and felodipine have been associated with approximately 12% lower risk of psychiatric and neurodegenerative outcomes than amlodipine, especially among women and individuals under 60 years of age without baseline psychiatric diagnoses [16]. In contrast, a population-based cohort of over 140,000 hypertensive patients revealed that CCB monotherapy was associated with nearly a twofold higher rate of mood-disorder admissions compared with renin–angiotensin system agents, even after multivariable adjustment [17].

Esketamine, approved for treatment-resistant depression, exerts secondary activity on L-type voltage-dependent calcium channels (L-VDCCs), reinforcing the relevance of calcium signaling in mood regulation [18]. Among newer dihydropyridines, isradipine demonstrated antidepressant efficacy in a pilot trial for bipolar depression, while nimodipine, when combined with fluoxetine, improved remission rates in unipolar and vascular depression [19,20,21].

These findings suggest that certain CCBs, especially those with central nervous system penetration, may offer adjunctive benefits in specific depressive subtypes. However, conflicting epidemiologic data and heterogeneous drug effects necessitate stratified clinical trials to clarify their role in MDD.

### 2.2. Bipolar Disorder and Schizophrenia

Beyond unipolar depression, CCBs have been explored as mood-stabilizing agents in bipolar disorder. Verapamil, nimodipine, and isradipine have demonstrated efficacy in small-scale trials, particularly for acute mania, supported by the observation that traditional mood stabilizers like lithium also exhibit calcium antagonism. CCBs offer potential clinical advantages such as minimal metabolic burden, limited sedation, and safety in pregnancy [22].

In schizophrenia, a nationwide Finnish cohort of over 60,000 individuals found that dihydropyridine use was associated with reduced psychiatric rehospitalization and all-cause mortality, a benefit not observed with non-dihydropyridine agents, likely due to differences in CNS penetration [23]. Results of a separate population analysis found fewer hospitalizations for affective symptoms among users of dihydropyridines and diltiazem, while verapamil showed benefit specifically in manic episodes and in patients under 40, suggesting age- and symptom-specific utility [24].

Recent pharmacogenetic evidence further supports this direction. A pilot study found that CACNA1B gene variants were associated with stronger antimanic responses to CCBs, suggesting a potential for personalized treatment [25]. Additionally, a small open-label trial of isradipine in schizophrenia patients receiving stable antipsychotic treatment demonstrated improvements in cognitive domains such as verbal memory and attention [26].

### 2.3. Attention-Deficit Hyperactivity Disorder (ADHD)

Recent findings suggest that amlodipine may offer therapeutic benefits in the management of attention-deficit hyperactivity disorder (ADHD). Amlodipine, a widely used dihydropyridine, crosses the blood–brain barrier and modulates neuronal excitability. Genetic variants in L-type calcium channel subunits (CACNA1C, CACNB1, and CACNA2D3) have been linked to ADHD susceptibility. UK Biobank data suggest that individuals with high genetic risk for ADHD who were treated with amlodipine reported fewer psychiatric symptoms, including mood instability and risk-taking behavior [27].

### 2.4. Neurodegenerative Diseases (NDs)

The large phase III STEADY-PD III trial aimed to determine whether isradipine can slow disease progression in patients with early-stage Parkinson’s disease. The final results showed no statistically significant difference between isradipine and placebo in slowing disease progression [28]. Despite negative results, the trial emphasizes the translational importance of calcium modulation in PD [28,29].

In a molecular dynamics simulation, nimodipine showed stable binding to MAOA with strong interaction energies, similar to the known MAOA inhibitor harmine. The results of the analysis indicated that nimodipine may affect the movement of important amino acid residues within MAOA and reduce its activity. Since MAOA overactivation is linked to neuronal damage in Alzheimer’s and Parkinson’s disease, this mechanism may contribute to nimodipine’s neuroprotective effects beyond its vascular actions [30].

Flunarizine has shown both therapeutic benefits and adverse effects in movement disorders, leading to conflicting interpretations of its role across neurological conditions. Epidemiologic studies report significantly increased risk of flunarizine-induced parkinsonism among migraine patients, particularly older individuals with comorbidities, raising concerns about its long-term antidopaminergic effects [31,32]. 

Conversely, case reports highlight its potential utility in certain genetic movement disorders. In rapid-onset dystonia-parkinsonism (RDP) linked to ATP1A3 mutations, complete symptom resolution was observed in a pediatric patient following flunarizine treatment [33]. In Alternating Hemiplegia of Childhood (AHC), also associated with ATP1A3 mutations, flunarizine significantly reduced the frequency and severity of paroxysmal attacks and has become the most commonly used symptomatic treatment [34].

These mixed results highlight how flunarizine’s effects can vary depending on individual genetic and clinical factors. Most of the current data come from case reports and observational studies, but the findings point to the need for more detailed research to better understand how flunarizine works in specific neurological conditions.

### 2.5. Epigenetic Considerations in CCB-Mediated Neuropsychiatric Effects

Although preclinical studies implicate epigenetic regulators like DNMT3A/B, Notch1, and MAPK14 in modulating L-type calcium channel expression relevant to stress and neurodevelopment [35,36], large-scale Mendelian randomization studies have failed to confirm a protective causal link between CCB-related genetic proxies and psychiatric disorders [37,38]. Results of a broader 2024 analysis found no protective signal and suggested possible risk elevation for bipolar disorder, major depression, and schizophrenia [39]. This mismatch is showing the need for mechanistic studies that can bridge molecular insights with clinical outcomes and potentially leverage epigenetic modulation as a therapeutic strategy.

The therapeutic potential of CCBs in neuropsychiatry may heavily depend on agent-specific properties (e.g., CNS penetration), patient-level factors (age, genetic variants), and underlying molecular mechanisms (calcium signaling, epigenetic regulation). To refine candidate selection and maximize translational value, multimodal approaches integrating pharmacogenomics, neuroimaging, and biomarkers should be prioritized.

## 3. Calcium Channel Blockers in Endocrine and Metabolic Disorders

### 3.1. Diabetes and Insulin Resistance

In pancreatic β-cells, voltage-gated calcium channels are essential for insulin release, as they allow calcium to enter the cell in response to glucose [40,41]. This calcium influx triggers insulin secretion. Some recent studies, especially with verapamil, suggest that CCBs may help protect β-cells and improve blood sugar control in both type 1 and type 2 diabetes. 

This section will review the mechanistic underpinnings and clinical evidence supporting the emerging role of CCBs in modulating β-cell health and metabolic outcomes.

#### 3.1.1. Effects on Pancreatic β-Cell Function

**Figure 2:**
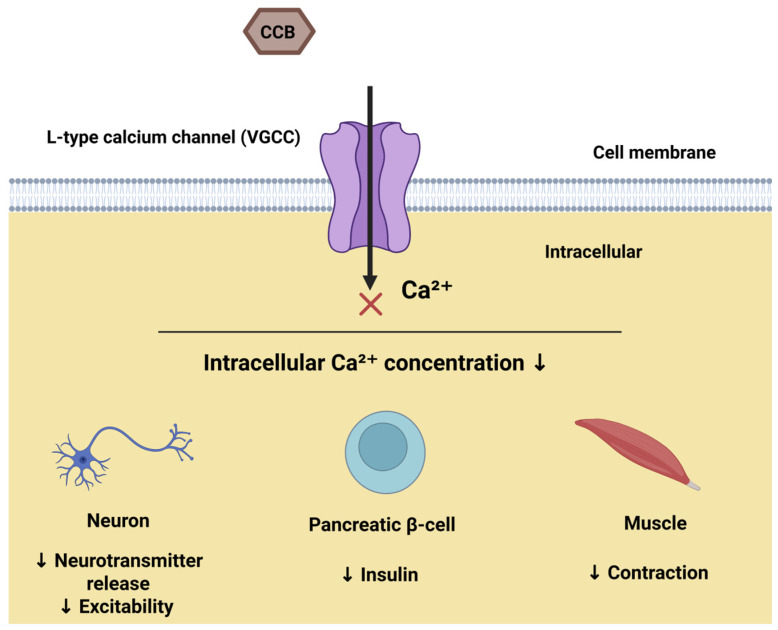
Mechanism of L-type calcium channel (VGCC) blockade by CCBs, leading to decreased intracellular Ca^2+^ concentration. Created in BioRender online application, available at https://app.biorender.com/. (Accessed on 18 June 2025).

Voltage-gated ion channels in the plasma membrane are critical for stimulus-secretion coupling in pancreatic β-cells. In particular, the influx of Ca^2+^ via voltage-gated calcium channels triggers exocytosis of insulin-containing granules [40,41]. These channels are activated by rhythmic membrane potential changes, initiated by glucose-induced closure of ATP-sensitive potassium (K-ATP) channels [41,42,43]. While sodium and potassium channels also influence β-cell function [41], calcium channels, specifically voltage-gated Cav3.2 and L-type Cav1.3, play central roles in glucose-induced electrical activity and insulin secretion. Exocytosis is primarily initiated by Ca^2+^ influx through P/Q-type (Cav2.1) channels [41]. Figure 2 illustrates this mechanism across different cell types.

Braun et al. demonstrated that blockade of L-type calcium channels with isradipine completely inhibits glucose-induced insulin secretion, highlighting the impact of CCBs on β-cell function [41]. 

Beyond insulin secretion, verapamil and other CCBs reduce the β-cell expression of thioredoxin-interacting protein (TXNIP), a proapoptotic factor overexpressed in diabetes, thereby inhibiting β-cell apoptosis and enhancing survival [44]. Clinical trials confirm verapamil’s beneficial effects on β-cells via downregulation of TXNIP and IGFBP3 and upregulation of IGF-1 signaling in type 1 diabetes (T1D) patients [45]. 

Chronic exposure to elevated free fatty acids (FFAs), especially saturated FFAs such as palmitic acid, disrupts β-cell Ca^2+^ homeostasis, inducing cellular stress and apoptosis [46,47,48]. Nifedipine, an L-type CCB, inhibits palmitate-stimulated Ca^2+^ release in rodent β-cell lines, reducing lipotoxicity and β-cell decompensation [46]. However, Šrámek et al. (2021) found that calcium influx inhibitors, including nifedipine and verapamil, did not mitigate saturated stearic acid–induced ER stress and apoptosis in human β-cell lines, suggesting limited benefit in preventing saturated fatty acid–induced β-cell apoptosis in humans [49]. Šrámek et al. did not assess effects on insulin secretion, leaving open the possibility that CCBs can protect β-cells by preserving insulin secretory function.

#### 3.1.2. Clinical Studies on CCBs and Metabolic Outcomes

A randomized, double-blind, placebo-controlled trial conducted in 2021 assessed the efficacy and safety of oral verapamil administration in patients with type 2 diabetes mellitus (T2DM) [46]. The study demonstrated a statistically significant reduction in HbA1c (~0.5%) in the verapamil-treated group by the end of the intervention period [50]. Although the decrease in TXNIP gene expression compared with placebo did not reach statistical significance, the findings suggest that verapamil may improve glycemic control in T2DM through downregulation of TXNIP expression and promotion of β-cell survival [50]. It is hypothesized that higher verapamil doses can achieve greater suppression of TXNIP, potentially enhancing therapeutic outcomes [10]. Comparisons of lipid profiles, including triglycerides, cholesterol, LDL, and HDL, as well as fasting blood glucose at study completion versus baseline revealed no significant differences between the verapamil and placebo groups [50]. 

A separate randomized, double-blind, placebo-controlled Phase 2 trial in 2018 demonstrated that adjunctive oral verapamil, when combined with standard insulin therapy, enhanced endogenous β-cell function and reduced exogenous insulin requirements and hypoglycemic episodes in adults with recent-onset type 1 diabetes (T1D) [51]. Subsequently, a 2022 Phase 2 randomized, double-blind, placebo-controlled clinical trial by Xu et al. revealed that continuous oral verapamil administration in T1D patients helps preserve β-cell function and diminishes the necessity for exogenous insulin, whereas discontinuation of verapamil therapy may accelerate disease progression [52], thus emphasizing its clinical relevance in T1D management. 

The notion of verapamil’s beneficial role in diabetes management is further supported by additional investigations. A retrospective, population-based cohort study utilizing Taiwan’s National Health Insurance Research Database (2000–2011) identified verapamil as uniquely effective among CCBs in reducing the risk of developing T2DM [53]. This association remained consistent across diverse subgroups and was especially notable in elderly populations [49]. Further evidence from analyses of the International Verapamil SR/Trandolapril (INVEST) trial indicated that participants receiving verapamil had a decreased incidence of diabetes onset [54,55]. Data from the Reasons for Geographic and Racial Differences in Stroke (REGARDS) study, a national cohort of community-dwelling middle-aged and older adults in the United States (2003–2007), showed that CCB use, particularly verapamil, correlated with lower fasting blood glucose levels among participants with diabetes [56]. Additionally, a self-controlled case series from 2021 found that concomitant use of CCBs with insulin secretagogues was associated with a reduced incidence of serious hypoglycemic events compared with insulin secretagogues alone, suggesting important clinical implications for cardiovascular drug selection in patients on insulin secretagogues [57].

In 2022, Wang et al. demonstrated that twice-daily oral administration of R-form verapamil (R-Vera), which confers enhanced β-cell survival and improved cardiovascular safety compared with racemic verapamil, combined with metformin, significantly improved glycemic control in T2DM patients by lowering HbA1c and fasting plasma glucose levels and enabled a greater proportion of patients to achieve target HbA1c levels below 7.0% [58].

Moreover, a multicenter randomized, double-blind clinical trial (Forlenza et al., 2023) conducted in children and adolescents newly diagnosed with T1D reported that verapamil partially preserved stimulated C-peptide secretion at 52 weeks postdiagnosis compared with placebo [59]. However, improvements in C-peptide levels were not accompanied by significant changes in glycated hemoglobin (HbA1c), blood glucose, or insulin requirements [59]. Despite this, the trial’s unique pediatric population highlighted the therapeutic potential of verapamil in preserving pancreatic β-cell function in this demographic [59,60].

Currently, a multicenter, randomized, double-blind, placebo-controlled trial (Ver-A-T1D) is underway in participants with T1D, investigating verapamil’s effect on the preservation of β-cell function. With final reporting anticipated in May 2026, this study aims to elucidate verapamil’s impact on β-cell thioredoxin-interacting protein expression in rodent and human islet cells and further clarify the role of CCBs in diabetes management [61].

### 3.2. Adrenal Disorders

#### 3.2.1. Role in Pheochromocytoma

Pheochromocytomas are tumors originating from catecholamine-producing chromaffin cells of the adrenal medulla, whereas paragangliomas arise from chromaffin cells located outside the adrenal glands. Collectively, these tumors are referred to as PPGL (pheochromocytoma and paraganglioma) [62,63]. 

Calcium channel blockers (CCBs) represent the most commonly used adjunct drug class to optimize blood pressure control in PPGL patients already receiving alpha-adrenergic receptor blockers [62,63]. According to several studies, CCBs serve three primary roles in managing pheochromocytoma. First, they supplement alpha-adrenergic blockade when blood pressure control remains insufficient, thereby avoiding escalation of alpha-blocker dosages. Second, CCBs may act as alternatives for patients experiencing severe adverse effects from adrenergic blockade. Third, they help prevent sustained hypotension caused by alpha blockers in patients exhibiting only intermittent hypertension [61,64]. In particular, Bravo et al. reported successful use of oral nifedipine capsules (10 mg) for managing hypertensive crises in pheochromocytoma [64].

Numerous investigations have considered CCB monotherapy as a potential alternative to alpha-blockade in preoperative pheochromocytoma management, with some authors proposing comparable efficacy [59,60]. These agents confer advantages such as reduced risk of overshoot hypotension, reflex tachycardia, and orthostatic hypotension, making them a safer option for normotensive patients experiencing paroxysmal hypertension or those intolerant to alpha blockers [62,65]. Additionally, CCBs have demonstrated cardioprotective effects, including prevention of myocarditis and coronary artery vasospasm [62,65].

Mechanistically, CCBs inhibit norepinephrine-mediated calcium influx into vascular smooth muscle cells rather than reducing catecholamine synthesis within tumors, thereby contributing to the control of hypertension and tachyarrhythmias [62,64,65].

Lebuffe et al. evaluated nicardipine as the preferred CCB for perioperative management during PPGL resection and found that although CCB monotherapy does not fully prevent all hemodynamic fluctuations, it is associated with low morbidity and mortality rates [66]. Results of a retrospective analysis demonstrated that nicardipine use during PPGL surgery yielded intraoperative hemodynamic stability and short-term postoperative outcomes comparable with those achieved with phenoxybenzamine [66]. Nicardipine’s selection as an alternative preoperative agent is based on its potent arterial vasodilatory effects without significant venous dilation, leading to afterload reduction and improved left ventricular function while maintaining venous return [66,67,68].

Brunaud et al. reported that patients with tumors ≥ 3 cm exhibited a higher incidence of hypotensive episodes under alpha-blocker therapy, whereas those receiving CCBs experienced more frequent hypertensive episodes [68]. In contrast, for tumors < 3 cm, no significant difference in intraoperative hemodynamic instability was observed between nicardipine and phenoxybenzamine treatment groups [67,68]. The most commonly employed CCBs in this context include nicardipine, amlodipine, and nifedipine [61,62].

#### 3.2.2. Role in Primary Aldosteronism

In 1985, Nadler et al. postulated that calcium channel blockade with nifedipine, both acutely and chronically, reduces plasma aldosterone levels in patients with primary aldosteronism caused by idiopathic hyperaldosteronism (IHA) or aldosterone-producing adenomas (APA) [69]. However, these findings were subsequently challenged by other authors [70,71]. Several commonly used dihydropyridine CCBs have been shown to compete with aldosterone for binding to the mineralocorticoid receptor (MR), acting as MR antagonists in addition to their L-type calcium channel blockade. They inhibit aldosterone-induced recruitment of coactivators and suppress transcription of MR target genes [72]. 

Despite this, in the majority of CCBs, the antihypertensive effect is primarily attributed to calcium channel blockade rather than MR antagonism. According to Deinum et al., most CCBs, including amlodipine, nitrendipine, nicardipine, and felodipine, do not achieve peak plasma concentrations at standard therapeutic doses sufficient to reach half-maximal inhibitory concentration (IC_50_) for the MR. Potential exceptions may include nifedipine and nimodipine [73]. Thus, Deinum et al. suggest it is unlikely that CCBs administered at standard antihypertensive doses are effective as monotherapy in counteracting MR-mediated aldosterone effects in primary aldosteronism [73]. Nonetheless, it has been hypothesized that certain CCBs may provide therapeutic benefit in patients harboring specific mutations in KCNJ5 and CACNA1D, which affect calcium influx. By inhibiting cytosolic calcium elevation essential for aldosterone synthesis, these agents may mitigate pathogenic effects associated with these mutations [74]. Although non-dihydropyridine L-type voltage-gated CCBs such as verapamil and diltiazem typically lack MR antagonistic activity, Tauber et al. demonstrated that these drugs strongly inhibit calcium channel activity in the presence of specific KCNJ5 mutations. Consequently, it has been proposed that these agents may reduce aldosterone synthesis and secretion when administered at sufficiently high doses in patients with these mutations [74,75]. A more recent study by Wang et al. (2020) reported that nifedipine, mibefradil, and benidipine significantly inhibit aldosterone secretion in patients with KCNJ5 mutations [76]. Furthermore, a 2020 case report confirmed the pathogenic role of CACNA1D in primary aldosteronism and demonstrated that nifedipine can effectively control blood pressure in patients carrying mutations in this gene [77].

Further research is required to clarify the role of CCBs in these specific patient populations and to better define their utility in clinical management.

## 4. CCBs in Pain Disorders

### 4.1. Calcium Channel Blockers in Neuropathic and Refractory Pain Treatment

Calcium channel blockers (CCBs) represent a key therapeutic class in neuropathic pain management through modulation of pathological neuronal excitability. While traditional L-type CCBs (e.g., verapamil) demonstrated limited efficacy in neuropathic pain, targeted agents acting on N-type (Cav2.2) channels (e.g., intrathecal ziconotide) and α2δ ligands (e.g., gabapentin, pregabalin) have emerged as first-line FDA-approved clinical treatments.

Pregabalin, a successor to gabapentin, is a first-line treatment for neuropathic pain, fibromyalgia, and epilepsy. It selectively binds to α2δ-1 and α2δ-2 subunits of VGCCs in the CNS. By modulating these subunits, pregabalin reduces the trafficking of VGCCs to presynaptic membranes and inhibits excessive calcium influx, thereby decreasing the release of excitatory neurotransmitters (e.g., glutamate, substance P, and norepinephrine) in hyper-excited neurons [78]. Unlike direct Cav2.2 blockers (e.g., ziconotide), pregabalin’s effects are state-dependent, preferentially targeting pathologically activated neurons while sparing normal function [79]. Clinical trials demonstrate efficacy in diabetic neuropathy, postherpetic neuralgia, and spinal cord injury pain, with dose-dependent relief [80]. However, central side effects (e.g., dizziness, somnolence) correlate with its CNS penetration, prompting development of α2δ-1-selective ligands (e.g., mirogabalin, crisugabalin) to improve tolerability.

Mirogabalin and crisugabalin (HSK16149) represent next-generation α2δ ligands designed to optimize neuropathic pain treatment. Mirogabalin exhibits prolonged binding to α2δ-1 and α2δ-2 subunits, with higher affinity for α2δ-1, enhancing analgesia in diabetic neuropathy and postherpetic neuralgia while reducing dosing frequency [81,82]. Crisugabalin demonstrates 23-fold greater α2δ-1 selectivity than pregabalin and a favorable pharmacokinetic profile, with 18-fold lower brain penetration in preclinical studies, minimizing CNS side effects like dizziness [83,84]. Both drugs maintain the core gabapentinoid mechanism: inhibiting presynaptic calcium currents and excitatory neurotransmitter release, but crisugabalin’s structural modifications further limit off-target effects with no abuse potential [85]. Phase III trials confirmed their efficacy in peripheral neuropathic pain, with crisugabalin approved in China for diabetic neuropathy and postherpetic neuralgia [85,86]. These agents exemplify targeted α2δ-1 modulation to improve tolerability and efficacy over pregabalin.

Ziconotide, a selective N-type (Cav2.2) calcium channel blocker, serves as a first-line intrathecal therapy for severe, refractory neuropathic pain. Its potent analgesic effects result from direct blockade of Cav2.2 channels in spinal cord dorsal horn neurons, which inhibits calcium influx and subsequent release of pro-nociceptive neurotransmitters (e.g., glutamate, substance P, and CGRP) from primary afferent nerve terminals [87,88]. Unlike opioids, ziconotide disrupts central pain transmission without inducing tolerance or respiratory depression, providing a valuable non-opioid alternative for managing conditions such as complex regional pain syndrome (CRPS), spinal cord injury-related pain, and refractory cancer pain [88]. Its inability to cross the blood–brain barrier (BBB) necessitates intrathecal administration; however, it does not prevent off-target systemic side effects on heart rate and blood pressure [87,88,89,90,91]. Ziconotide’s clinical use faces significant limitations due to its invasive intrathecal administration requiring risk of procedure complications, narrow therapeutic window necessitating careful dose titration, and delayed onset of action (1–2 weeks) limiting utility in acute pain crises [87,89]. Additionally, standard contraindications for intrathecal therapy apply, including active infections at the injection site, spinal abnormalities that impede cerebrospinal fluid flow, or uncontrolled bleeding disorders [87,88]. Treatment is frequently interrupted by dose-dependent adverse effects, including cognitive and neuropsychiatric disturbances (e.g., ataxia, depression, suicidal ideation, and hallucination), meningitis, and creatine kinase elevation. These safety concerns necessitate dose adjustments and contraindicate use in patients with a history of psychosis, major psychiatric disorders, or renal impairment [87,88]. These constraints, combined with the need for intensive monitoring, restrict its use to refractory cases under close supervision. Future development is dependent on the ability to improve bioavailability and reduce off-target/peripheral effects while maintaining central N-type calcium channel blockade [92]. 

A meta-analysis study of 81 randomized controlled trials (RCTs) involving 10,003 patients compared non-opioid and opioid therapies for chronic cancer pain management. The study ranked ziconotide as the most effective treatment for reducing pain intensity and pregabalin for global efficacy, currently positioning CCBs among the most effective medications in pain therapeutics while maintaining favorable safety profiles [93]. 

### 4.2. Migraine and Chronic Headache

Calcium channel blockers (CCBs) occupy a unique niche in migraine management, with flunarizine and verapamil representing distinct therapeutic approaches that target both vascular and neuronal components of migraine pathophysiology. Flunarizine, a nonselective CCB with additional sodium channel modulation, demonstrates dual mechanisms in migraine prophylaxis: it suppresses cortical spreading depression (CSD) (the electrophysiological correlate of migraine aura) while stabilizing trigeminovascular activation through the inhibition of calcitonin gene-related peptide (CGRP) release [94,95]. Clinical studies show that flunarizine achieves a ≥50% reduction in migraine frequency, intensity, and duration in many patients, with biomarker studies revealing a 478% increase in serotonin (5-HT) and decreased CGRP levels after eight weeks of treatment [96]. Despite its classification as a first-line prophylactic in Europe with efficacy comparable with propranolol in large trials (*n* = 808 and *n* = 400), debates persist regarding the quality of older studies, prompting calls for contemporary randomized controlled trials to confirm its place in therapy [97]. Intriguingly, flunarizine’s neuroprotective effects extend beyond migraine, with recent evidence showing benefit in sudden sensorineural hearing loss (SSNHL), a condition sharing similar vascular and excitability mechanisms [98].

Verapamil exerts stronger vascular effects through cerebral vasodilation, which makes it particularly valuable for cluster headache prophylaxis, though its efficacy in migraine prevention remains more modest [99]. These differential effects underscore the complex interplay between vascular (L-type) and neuronal (T-type/N-type) calcium channels in headache disorders. While newer targeted therapies like CGRP monoclonal antibodies (e.g., gepants, ditans) have revolutionized acute migraine treatment [100], CCBs such as nimodipine retain clinical value due to their broader modulation of migraine pathophysiology and neurovascular protection [13]. Emerging evidence of shared vascular–neuronal mechanisms between migraine and cardiovascular disease further reinforces the potential of CCBs in comorbid populations, particularly in patients with aura or comorbid vascular risk factors [13,101]. 

Future research directions include the development of next-generation CCBs with enhanced selectivity for neuronal channels involved in CSD and trigeminovascular activation, as well as combination strategies with CGRP-targeted therapies for refractory cases. The continued investigation of biomarkers (such as 5-HT, BDNF, CGRP, etc.) may further personalize CCB therapy, optimizing outcomes for distinct migraine subtypes.

### 4.3. Neuropathic, Visceral, and Inflammatory Pain: Therapeutic Potential of Calcium Channel Blockers in Development

#### 4.3.1. N-Type (Cav2.2) Antagonists 

The clinical utility of N-type CCBs has been constrained by challenges in balancing efficacy and safety. While ziconotide remains the gold standard for intrathecal use in refractory pain, its invasive administration limits broader application. Recent efforts to develop oral Cav2.2 antagonists (e.g., CNV2197944, Z160) have struggled to replicate ziconotide’s efficacy without incurring gabapentinoid-like central side effects, leading to Phase II trial failures for neuropathic pain [90,102]. These setbacks underscore the need for improved subtype selectivity and pharmacokinetic optimization in next-generation N-type blockers.

PRI-002 (previously RD2) is a novel N-type (Cav2.2) calcium channel modulator designed to overcome ziconotide’s limitations by combining BBB penetration with a favorable safety profile [91,102]. Unlike ziconotide, RD2 can be systemically administered (oral or intravenous) while still targeting spinal and supraspinal pain pathways, as demonstrated by its efficacy in preclinical neuropathic pain models [91]. Phase I trials confirmed its tolerability without ziconotide-like cardiovascular side effects, though analgesic efficacy in humans remains unproven [103]. A key advantage is its nonpeptide structure, which avoids the stability and delivery challenges of peptide-based blockers. However, its moderate Cav2.2 selectivity raises concerns about potential off-target effects at higher doses or failure to show translational efficacy in human trials, necessitating careful dose optimization moving forward.

CC2230 is a next-generation, state-dependent N-type (CaV2.2) calcium channel blocker that selectively inhibits hyperactive nociceptive pathways while preserving baseline neuronal function. In preclinical studies, CC2230 demonstrated robust efficacy across multiple pain models (including neuropathic, orofacial, and osteoarthritis pain) when administered systemically, intrathecally, or intranasally, with the intranasal route showing particular potential for treating refractory trigeminal pain conditions [102]. Mechanistic studies revealed CC2230′s unique ability to preferentially bind inactivated CaV2.2 channels, resulting in use-dependent inhibition that targets pathological pain signaling without inducing tolerance or motor impairment: a critical advantage over previous failed N-type blockers [90,102]. Molecular docking and mutagenesis studies have further characterized CC2230′s binding site, enabling rational optimization for enhancing specificity and determining exact mechanism [90,102]. While its differentiated pharmacokinetic profile and multiple administration routes address key limitations of earlier candidates, clinical validation will be essential to determine whether CC2230 can successfully translate its promising preclinical profile into therapeutic breakthroughs for chronic pain management.

#### 4.3.2. T-Type (Cav3.2) Antagonists

Cav3.2 T-type calcium channels serve as key molecular amplifiers in visceral and inflammatory pain pathologies [104,105]. In visceral pain disorders such as irritable bowel syndrome and chronic pancreatitis, Cav3.2 channels serve as critical amplifiers of nociceptive signaling with upregulated expression in dorsal root ganglia and spinal cord neurons contributing to the characteristic poorly localized discomfort [105,106]. Inflammatory pain (IP) results from tissue damage or immune activation (e.g., colitis, arthritis), driving prostaglandin and cytokine release that sensitizes peripheral nociceptors [107]. Similarly, inflammatory mediators (e.g., reactive oxygen species) exploit redox-sensitive modulation of Cav3.2 to sustain peripheral and central sensitization across pain subtypes, making it a compelling therapeutic target [108]. 

Cav3.2 T-type calcium channels exhibit widespread expression patterns across multiple physiological systems, with significant presence in both central and peripheral nervous systems. These channels are functionally important in diverse tissues including the cochlear hair cells (auditory processing), vascular and smooth muscle (vasoregulation), pancreatic β-cells (insulin secretion), and adrenal medulla (catecholamine release), where they modulate distinct but frequently interrelated physiological functions [109].

The broad therapeutic potential of Cav3.2 modulation is exemplified by ML-218′s development pathway. This brain-permeable T-type channel blocker has shown the following: antineoplastic effects in oral cancer models by disrupting calcium-dependent proliferation pathways [110], efficacy in Parkinson’s disease paradigms through modulation of subthalamic nucleus neuronal activity [111], analgesic properties in neuropathic pain and hypersensitivity models via peripheral and central mechanisms [112], and more. Emerging evidence reported that ML-218 treatment can substantially reduce key markers of neuroinflammation (e.g., TNF-α, IL-6) and oxidative stress (e.g., ROS) while improving functional recovery in rodent stroke models. These results position ML-218 as a promising repurposing candidate for ischemic stroke, demonstrating robust neuroprotective effects through anti-excitotoxicity mechanisms in preclinical models while leveraging its established safety profile from prior pain trials [113].

This multi-indication potential stems from Cav3.2′s fundamental role in regulating cellular excitability and calcium signaling across different tissue types. Preclinical studies consistently demonstrate that Cav3.2 antagonists successfully normalize aberrant neuronal firing patterns and attenuate visceral hypersensitivity and inflammatory pain behaviors, while preserving acute nociception [114,115]. However, the channel’s widespread distribution also presents challenges for targeted therapy, necessitating careful consideration of tissue-specific delivery methods and potential off-target effects in clinical development. 

ABT-639, a highly selective, peripherally restricted Cav3.2 antagonist, demonstrated significant preclinical efficacy in inflammatory and neuropathic pain models. Rodent studies displayed its ability to reduce dorsal root ganglion (DRG) neuron sensitization and increase tactile allodynia thresholds with tolerable safety profile, achieving 70–80% target occupancy with over 100-fold selectivity over other calcium channel subtypes [116]. Despite this achievement, ABT-639 failed to meet primary endpoints in Phase II trials for diabetic neuropathy, with pharmacokinetic limitations including low oral bioavailability at the maximum tolerated dose and narrow therapeutic window likely contributing to insufficient target engagement at clinically achievable concentrations [110,111]. Post hoc analyses suggested efficacy in subgroups with confirmed Cav3.2 upregulation, highlighting the need for biomarker-driven stratification [117]. T-type calcium channels are promising targets for pain management, but peripherally restricted antagonists have clinically failed, suggesting that central inhibition may be critical.

Conversely, Ulixacaltamide (formerly Z944), a CNS-penetrant T-type blocker, boasts a 100-fold greater potency than ABT-639 and provides pain relief upon intrathecal delivery in both neuropathic and inflammatory pain. Its mechanism effectively suppresses spinal lamina I neuron excitability, which plays a large role in pain perception, and reverses inflammatory pain through inhibition of low-threshold T-type currents [114]. However, Phase I trials revealed dose-limiting sedation and dizziness, stalling further development pending the modification of formulation [118]. Despite this, Z944 validated central T-type modulation as a viable therapeutic strategy [114]. 

The failure of purely peripheral (ABT-639) and broadly central (Z944) T-type blockers highlights the need for balanced targeting. Significant challenges remain in translating these promising preclinical results to clinical efficacy in humans, particularly regarding optimal drug delivery and patient stratification, and overcoming species-specific differences in channel pharmacology [119].

### 4.4. Challenges and Emerging Directions

The limitations of first-generation CCBs in pain have spurred the development of innovative approaches to pain management, particularly dual-target inhibitors. Current research is elucidating endogenous Cav3.2 modulators (e.g., serum peptides) and channel interaction domains to enable novel targeting strategies, including gene therapies and monoclonal antibodies [104]. Among promising candidates in discovery-phase studies is MONIRO-1, a dual Cav2.2/Cav3.2 inhibitor with blood–brain barrier permeability for neuropathic pain [120]. Another molecule, CNCB-2, acts as a cationic dual modulator of Nav1.7 and Cav2.2, demonstrating efficacy in hyperalgesia and allodynia in ocular pain [121], incision wound, and inflammatory pain, with rapid onset extracellular application comparable with lidocaine [122]. Additionally, dual blockade of Cav3.2 and TRPV1 channels shows strong potential in neuropathic pain by simultaneously addressing calcium-mediated neuronal hyperexcitability and neurogenic inflammation, potentially reducing effective dosages [123,124]. These approaches aim to overcome the historical trade-off between target engagement and tolerability seen in earlier candidates.

The clinical development of CCBs has increasingly pivoted toward neuropsychiatric indications, as seen with Z944′s ongoing Phase III evaluation for essential tremor and PRI-002′s ongoing Phase II trials for Alzheimer’s disease. This shift has created knowledge gaps in pain applications. Nevertheless, cutting-edge research persists in exploring novel calcium channel modulators and advanced delivery systems using cryo-EM-guided docking and AI-driven simulations [104]. Current efforts prioritize CNS-sparing, state-dependent antagonists and nanoparticle-enabled delivery systems to mitigate long-standing pharmacokinetic challenges, including poor bioavailability and off-target effects.

The translational hurdles experienced by existing CCBs (both marketed and in development) inform the development of next-generation therapies. Calcium channel antagonists remain compelling candidates for pain due to their dual modulation of neuronal hyperexcitability and inflammatory pathways. However, their successful clinical translation will require further understanding of biomarker-driven patient stratification, combinatorial regimens with complementary targets (e.g., Nav1.8 or CGRP inhibitors), and re-repurposing of neuropsychiatric candidates for pain indications.

## 5. Regulatory and Translational Considerations in CCB Repurposing

The repositioning of CCBs for noncardiovascular indications faces significant regulatory and commercial hurdles. Patent limitations and generic availability of older CCBs reduce incentives for costly Phase III trials, despite their therapeutic potential in niche populations [125,126]. While drug repurposing offers theoretical advantages in development timelines and costs by circumventing early-phase safety testing, these benefits are frequently negated by intellectual property challenges and the uncertain success of late-stage clinical trials, creating significant disincentives for potential investors [126,127,128].

This has spurred interest in novel formulations and delivery systems (e.g., nanoparticle-encapsulated dihydropyridines [129], microneedle transdermal delivery of ziconotide [130], etc.) to secure intellectual property and improve pharmacokinetics [131]. Advances in computational approaches (e.g., drug-disease mapping using signature matching [126], AI-driven molecular predictions [132], etc.) may facilitate patentable reformulations or discover repositioning opportunities. Combined with regulatory mechanisms like the FDA’s 505(b)(2) pathway, these technological advances provide crucial frameworks for leveraging existing safety data while enabling patentable innovations [126,127,128]. The repurposing of existing drugs needs careful evaluation of both ethical obligations and patient safety concerns. Main issues include maintaining FDA-mandated clinical trial transparency, establishing standardized protocols for off-label use, and preventing premature clinical adoption. The CLVer trial [133]. illustrates these challenges, showing verapamil’s potential for β-cell preservation in new-onset type 1 diabetes while highlighting critical limitations including small sample size, intensive monitoring requirements, and unoptimized dosing regimens. Although off-label use represents a well-established clinical practice, premature adoption of repurposed therapies carries significant risks: undefined safety margins with unvalidated dosing schedules, increased medicolegal liability, and potential patient harm from unexpected adverse effects [126,134,135].

Real-world evidence from pragmatic trials and large-scale observational studies plays a pivotal role in CCB repurposing efforts. For example, post hoc analyses of completed clinical trial data (e.g., cardiovascular trial data repurposed for diabetes research [60]) and nationwide pharmacoepidemiologic studies (e.g., CCBs in bipolar disorder [24]) reveal potential therapeutic associations through population level prescribing patterns and outcomes, while addressing evidence gaps from often underpowered, off-patent clinical trials. These approaches are particularly valuable for identifying both efficacy signals and safety concerns in diverse patient populations, informing subsequent controlled investigations [24,60].

To balance therapeutic innovation with patient protection, regulatory agencies must enforce rigorous trial disclosure requirements and develop evidence-based monitoring guidelines for off-label use.

## 6. Conclusions

This review underscores the expanding therapeutic potential of CCBs beyond their established cardiovascular applications, highlighting their roles in neuropsychiatric, endocrine, and pain disorders. In neuropsychiatric disorders, dihydropyridines like nimodipine and isradipine demonstrate promise in bipolar disorder, vascular depression, and schizophrenia, though conflicting epidemiological data and heterogeneous trial outcomes necessitate further stratified studies to clarify their efficacy and safety. The neuroprotective potential of CCBs in neurodegenerative diseases remains biologically plausible but clinically unproven, as evidenced by the neutral results of isradipine in Parkinson’s disease.

In endocrinology, verapamil has emerged as a compelling candidate for preserving β-cell function in diabetes, with clinical trials confirming its ability to reduce insulin requirements and hypoglycemic episodes in type 1 diabetes. However, the translational impact of other CCBs in metabolic syndromes and adrenal disorders remains limited by inconsistent evidence, with select agents like nifedipine showing utility only in specific genetic subtypes of primary aldosteronism. For pain disorders, CCBs exhibit divergent profiles: while α2δ ligands and N-type blockers are well established for neuropathic pain, newer agents like mirogabalin and crisugabalin aim to improve tolerability through subunit selectivity. T-type Cav3.2 antagonists, though promising in preclinical models, face translational challenges due to bioavailability constraints and off-target effects, underscoring the need for advanced delivery systems and biomarker-driven patient selection.

Moving forward, the successful repurposing of CCBs will depend on addressing key limitations: (1) optimizing blood–brain barrier penetration and tissue selectivity to enhance CNS efficacy while minimizing peripheral adverse effects; (2) elucidating pharmacogenetic predictors of response to enable precision medicine approaches; and (3) prioritizing combinatorial strategies, such as pairing CCBs with CGRP inhibitors in migraine or Nav1.7 blockers in neuropathic pain. Future trials should incorporate standardized outcome measures, longer follow-up periods, and mechanistic biomarkers to validate hypotheses generated from preclinical models. As the field advances, the integration of cryo-EM, AI-driven drug design and targeted nanoparticle delivery may overcome historical barriers, ushering in a new era of calcium channel therapeutics with expanded clinical utility.

## Data Availability

No new data were created or analyzed in this study.
